# Acute transient freezing of gait in a patient with posterior reversible encephalopathy syndrome

**DOI:** 10.1186/1471-2377-13-79

**Published:** 2013-07-09

**Authors:** Asuka Nakajima, Yuji Ueno, Hideki Shimura, Taiki Kambe, Kenya Nishioka, Nobutaka Hattori, Takao Urabe

**Affiliations:** 1 Department of Neurology, Juntendo University Urayasu Hospital, 2-1-1 Tomioka, Urayasu, Chiba 279-0021, Japan; 2 Department of Neurology, Juntendo University School of Medicine, 2-1-1 Hongo, Bunkyo, Tokyo 113-8421, Japan

**Keywords:** Posterior reversible encephalopathy syndrome, Freezing of gait, Parkinsonism, Vasogenic edema, Hemodialysis

## Abstract

**Background:**

Posterior reversible encephalopathy syndrome (PRES) is a transient clinical and neuroradiologic syndrome caused by cerebral vasogenic edema. Various reversible neurologic symptoms were shown in patients with PRES. Freezing of gait (FOG) is mainly observed in neurodegenerative diseases.

**Case presentation:**

We report a 43-year-old man, with undergoing hemodialysis therapy for chronic renal failure, had mild elevation of blood pressure. His consciousness level suddenly deteriorated, and brain MRI demonstrated hyperintense lesions in the bilateral basal ganglia on fluid-attenuated inversion recovery images, diffusion-weighted images, and apparent diffusion coefficient maps. After improvement of disturbance of consciousness, he showed FOG accompanied by bradykinesia and postural instability. His FOG spontaneously improved concurrently with alleviation of basal ganglionic lesions on follow-up MRI.

**Conclusions:**

It is suggested that vasogenic edema on bilateral basal ganglia associated with PRES can cause acute transient FOG.

## Background

Posterior reversible encephalopathy syndrome (PRES) is usually described as presenting with a variety of reversible neurologic symptoms including headache, seizures, vision loss, and deterioration of consciousness [1,2]. Vasogenic edema caused by a breakdown of autoregulation of cerebral blood flow is proposed as a principal pathogenesis of PRES [1,2]. For the diagnosis of PRES, increasing signal intensity on apparent diffusion coefficient maps (ADC maps) are indispensable to differentiate vasogenic edema from cytotoxic edema, although diffusion-weighted images (DWI) and fluid-attenuated inversion recovery (FLAR) images might show high signals in both vasogenic and cytotoxic edemas.

Freezing of gait (FOG), often accompanied by bradykinesia, rigidity, and postural instability, is observed in several neurological disorders including Parkinson’s disease and parkinsonian syndromes [3,4].

Here, we report a 43-year-old man who suddenly developed FOG associated with vasogenic edema in the bilateral basal ganglia caused by PRES. His FOG spontaneously improved concurrently with alleviation of basal ganglionic lesions. It is suggested that vasogenic edema on bilateral basal ganglia associated with PRES can cause transient FOG.

## Case presentation

**Figure 1:**
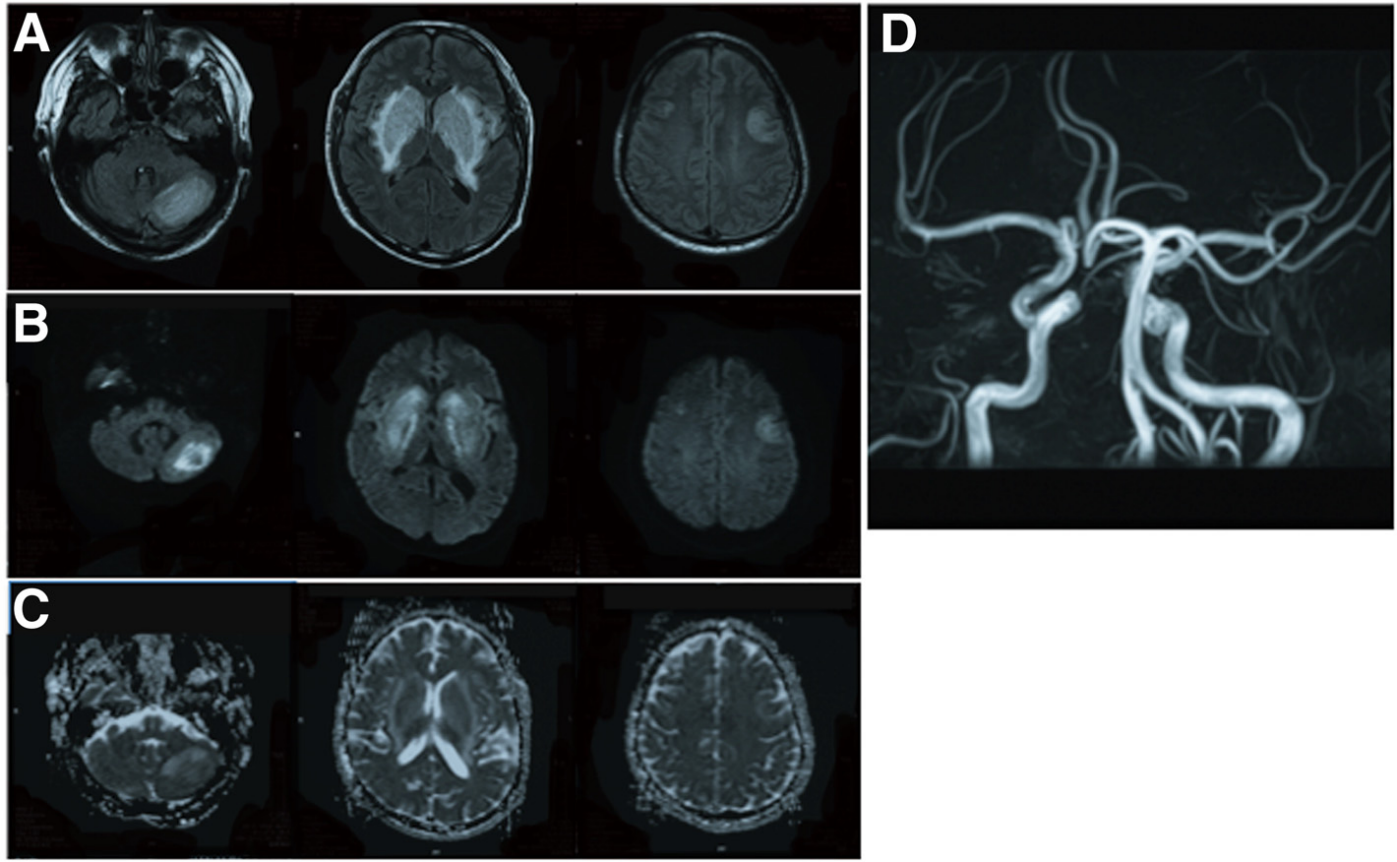
**MR images on admission.** Representative images of brain MRI including fluid attenuated inversion recovery (FLAIR) **(A)**, diffusion-weighted image (DWI) **(B)**, apparent diffusion coefficient (ADC) map **(C)**, and MR angiography **(D)** on admission, showing high-signal-intensity lesions in the bilateral basal ganglia, left cerebellar hemisphere, and bilateral frontal cortices.

**Figure 2:**
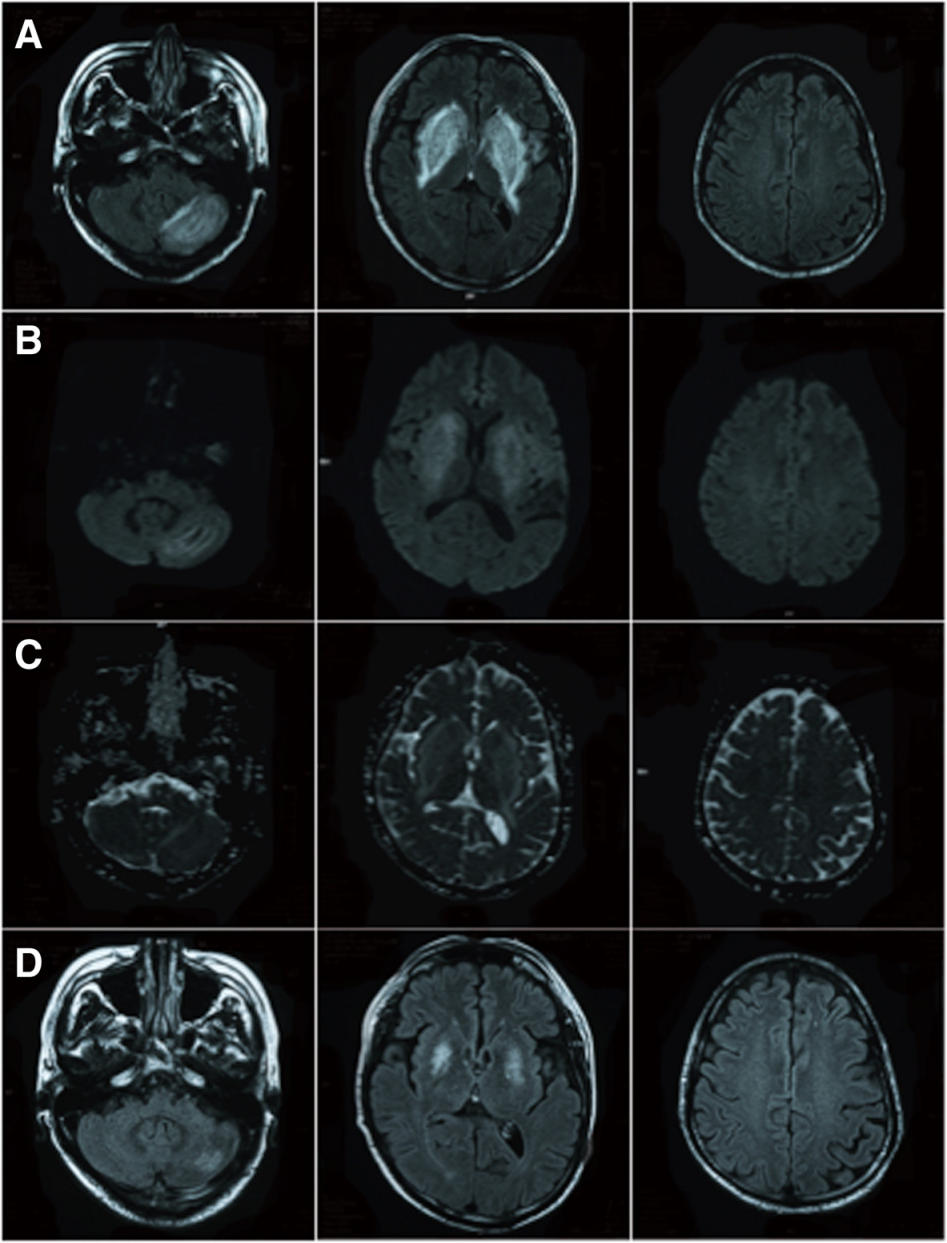
**MR images at 1 and 4 weeks after admission.** Representative FLAIR images **(A)**, DWI **(B)**, and ADC map **(C)** at 1 week after admission, and FLAIR images **(D)** at 4 weeks after admission.

A 43-year-old man, who had undergone hemodialysis therapy for chronic renal failure owing to diabetic nephropathy for 6 years and whose blood pressure had been under control (<140/80 mmHg), developed mild elevation of blood pressure (150/100 mmHg) for 2 weeks. He has not been treated with immunosuppressant agents. He suddenly showed weakness in both of his legs, and he realized that he had postural instability and that he could not walk. Subsequently, his consciousness level deteriorated. On admission, his blood pressure was 156/96 mmHg. Neurological examination showed a Glasgow Coma Scale of 14(E3V5M6). His facial expression was flat and his voice was barely audible. He had paraparesis in both lower limbs. Brain MRI demonstrated hyperintense lesions in the bilateral basal ganglia, bilateral frontal cortices, and left cerebellum on FLAIR images, and those lesions were hyperintensity on DWI and ADC maps (Figure 1A–C). No arterial stenosis on MR angiography was found (Figure 1D). He was treated with anti-hypertensive agents and continued hemodialysis therapy, and his blood pressure were controlled to 130/70 mmHg within 2 weeks. On the 7th day after admission, disturbance of consciousness and paraparesis improved, and he could walk by himself. However, we noticed that he showed parkinsonian signs including FOG, bradykinesia, and postural instability. His FOG fulfilled the criteria of “brief, episodic absence or marked reduction of forward progression of the feet despite the intention to walk” based on the 2010 workshop of clinicians and scientists interested in FOG [5]. The score from part III of the Unified Parkinson’s Disease Rating Scale (UPDRS) was 28 points. He did not show cerebellar ataxia. Concurrently, MRI showed hyperintense basal ganglionic lesions on FLAIR images, DWI, and ADC maps persisted, although lesions on bilateral frontal cortices were diminished (Figure 2A-C). His FOG and postural instability gradually improved, and UPDRS part III scores were 22 and 5 points at 2 and 4 weeks after admission, respectively. Follow-up MRI at 4 weeks after admission showed significant alleviation of the bilateral basal ganglionic lesions on FLAIR (Figure 2D). Thus, he was diagnosed as PRES based on the acute transient neurological symptoms and MRI findings. He was treated with 150 mg of amantadine, and a combination of 300 mg of levodopa and 30 mg of carbidopa at 15th and 22st day after admission for 7 days, respectively, but he did not show dramatic improvement of FOG. Thereafter, those anti-parkinsonian drugs were discontinued.

## Discussion

The basal ganglia is involved in 12% to 34% of patients with PRES, and is especially common in patients with preeclampsia-eclampsia [1,2]. Compared to the cortical gray matter, the basal ganglia contains a large number of non-anastomotic vessels and capillary beds, and may therefore be more vulnerable to increased systemic blood pressure and circulating toxins [6]. While hemodialysis is generally used for the treatment of renal disease, hemodialysis itself may cause vascular endothelial damage [7]. Thus, we concluded that endothelial damage caused by elevation of blood pressure and hemodialysis might be closely associated with vasogenic edema in the bilateral basal ganglia, which resulted in PRES in our case. To the best of our knowledge, we firstly reported that vasogenic edema on bilateral basal ganglia associate with PRES could cause acute parkinsonian freezing gait, and that alleviation of basal ganglionic lesions was consistent with an improvement of his FOG.

FOG and postural instability are thought to be manifestations of dysfunction of pedunculopontine nucleus (PPN) and mesencephalic locomotor region (MLR), which are terminals with gamma-aminobutyric acid-ergic (GABAergic) output that are regulated by the globus pallidus internus (GPi) and substantia nigra pars reticulata (SNr) [8]. Previously, abnormal signal intensity in GP, putamen, dorsolateral SN, and PPN on MRI were shown in patients with neoplasm, cerebrovascular disease, and CO intoxication who developed FOG [9-11]. Thus, disorder of GP, putamen, dorsolateral SN, and PPN can cause FOG. Although the precise pathophysiology underlying FOG remains unclear, we suggest that extensive vasogenic edema in the bilateral striatum reduced inhibition of GPi/SNr, and led secondarily to activation of the GPi/SNr and suppression of the PPN/MLR, thereby resulting in FOG and postural instability in the present case.

For patients with hypertension and undergoing hemodialysis, who suddenly develop FOG, physicians should be aware of the possibility of bilateral basal ganglionic lesions and PRES.

## Conclusions

It is suggested that vasogenic edema on bilateral basal ganglia associated with PRES can cause acute transient FOG.

### Patient consent

Written informed consent was obtained from the patient for publication of this case report and any accompanying images. A copy of the written consent is available for review by the Editor-in-Chief of this journal.

## Competing interest

The authors declare that they have no competing interests

## Authors’ contributions

Acquisition of data: AN and YU. Analysis and interpretation of data: AN, YU, HS, TK, and KN. Drafting of the manuscript: AN, YU, HS, and TU. Critical revision of the manuscript for important intellectual content: YU, NH, and TU. All authors read and approved the final manuscript.

## Pre-publication history

The pre-publication history for this paper can be accessed here:

http://www.biomedcentral.com/1471-2377/13/79/prepub
